# Prognostic Factors of the Progression of Chronic Kidney Disease and the Development of End-Stage Renal Disease in Patients with Lupus Nephritis: A Retrospective Cohort Study

**DOI:** 10.3390/jcm14030665

**Published:** 2025-01-21

**Authors:** Bianka Perge, Gábor Papp, Bernadett Bói, Csilla Markóth, László Bidiga, Nikolett Farmasi, József Balla, Tünde Tarr

**Affiliations:** 1 Division of Clinical Immunology, Institute of Internal Medicine, Faculty of Medicine, University of Debrecen, H-4032 Debrecen, Hungary; pergebianka@med.unideb.hu (B.P.); papp.gabor@med.unideb.hu (G.P.); farmasi.nikolett@med.unideb.hu (N.F.); 2 Department of Public Health and Epidemiology, Faculty of Medicine, University of Debrecen, H-4028 Debrecen, Hungary; boi.bernadett@med.unideb.hu; 3 Division of Nephrology, Institute of Internal Medicine, Faculty of Medicine, University of Debrecen, H-4032 Debrecen, Hungary; markoth.csilla@med.unideb.hu (C.M.); balla@belklinika.com (J.B.); 4 Institute of Pathology, Faculty of Medicine, University of Debrecen, H-4032 Debrecen, Hungary; bidiga.laszlo@med.unideb.hu

**Keywords:** lupus nephritis, chronic kidney disease, end-stage renal disease, complete remission, chronicity index

## Abstract

**Background/Objectives**: Lupus nephritis (LN) is one of the most severe organ manifestations of systemic lupus erythematosus (SLE). Chronic kidney disease (CKD) and its progression into end-stage renal disease (ESRD) are serious complications in LN and the main cause of death in SLE. We aimed to investigate the prognostic factors of the progression of CKD and the development of ESRD in SLE patients. **Methods**: In our retrospective cohort study, we assessed the clinical and laboratory data of 127 patients who were diagnosed with LN between 1990 and 2022 and received regular follow-up care at our autoimmune centre. We compared class IV (diffuse) LN patients with non-class IV LN patients and assessed the differences in clinical and laboratory data of the patients, subdivided into complete, partial, and non-responders to therapy. **Results**: The prevalence of class IV LN is significantly higher in patients with CKD stage 3–5. Age above 42, class IV LN, Coombs positivity, and high chronicity index are prognostic factors for the development of CKD stage 3–5. On the other hand, anti-RNP and anti-SS-B antibody positivity and a high chronicity index are prognostic factors for the development of ESRD. The chronicity index, as well as the SLICC/ACR Damage Index (SDI) score, was significantly higher in non-responders compared to patients with complete remission. **Conclusions**: Based on our results, the progression of CKD into stage 3–5 or the development of ESRD should be expected at a chronicity index above 3.5 points. An early diagnosis, as well as aggressive, timely, and adequate treatment, is fundamental to prevent unfavourable outcomes of LN.

## 1. Introduction

Lupus nephritis (LN) is one of the most severe organ manifestations of systemic lupus erythematosus (SLE). Almost all patients with SLE have renal involvement during the course of the disease, but manifest lupus nephritis develops in 40–70% of patients [1,2]. Based on a recent review of the literature, LN may be the first manifestation of SLE in 7–31% of cases, and it develops in 31–48% of patients during the course of the disease [3]. In most cases, LN appears in the first five years of the disease, after which its development is rare [4]. As lupus nephritis implies persistent alterations to kidney structures and functions, all patients with lupus nephritis have chronic kidney disease (CKD) [5]. There has been a rapid advancement in our understanding of the pathogenesis of LN. In genetically susceptible individuals, an immune-complex-mediated glomerulonephritis develops with the contribution of certain epigenetic factors. Among the clinical signs, the appearance of proteinuria, active urine sediment, decreased renal function, nephrosis, and nephritis syndrome indicate glomerular damage. At the same time, not only can the glomeruli be involved, but sometimes, interstitial and tubular damages occur, or thrombotic microangiopathy develops, which can also contribute to renal impairment. The degree of proteinuria is influenced by the destruction of podocytes [4,6]. The gold standard for the diagnosis of LN is a renal biopsy. In all cases, if lupus nephritis is suspected, a renal biopsy is recommended, which helps in the differential diagnosis of other glomerular diseases, as well as with determining the patient’s treatment. It also has a prognostic value [7,8]. LN is classified into six histological categories, which were classified by the WHO and then revised twice by the ISN/RPS, leaving the basic histological types intact [9,10,11]. The class IV LN, namely diffuse proliferative glomerulonephritis, is the most common histological type and is considered a poor prognostic factor. The progression of CKD and the development of end-stage renal disease (ESRD) are the most prevalent characteristics in this category [12,13]. The introduction of cyclophosphamide was a milestone in the therapy of LN and significantly improved the survival of patients [14]. In the last two decades, significant advances have been made in the therapy of LN [15]. The use of mycophenolate mofetil has been introduced in both induction and maintenance treatment. A new calcineurin inhibitor, voclosporin, has been registered in combination with MMF in the treatment of LN. Belimumab treatment in lupus nephritis is also based on an accepted indication in combination with cyclophosphamide, azathioprine, and MMF. Rituximab may also be administered in therapy-refractory cases [16,17,18,19,20]. To follow the course of the disease and measure the effectiveness of the therapy, one option is a repeated renal biopsy, but this cannot be a routine procedure. An alternative method in everyday practice is to measure certain serum and urine biomarkers. The quantity of proteinuria, examination of urine sediment, ratio of urine total protein/creatinine, estimated glomerular filtration rate (eGFR), and anti-double-stranded DNA (anti-dsDNA) antibodies are the most commonly monitored parameters. However, these parameters are not specific and sensitive enough for calculating the renal activity and damage [3]. A number of new urine and serum biomarkers have been investigated that may help in the monitoring of renal disease, but they are not yet available in daily practice [21,22]. Therefore, clinical observations and currently available laboratory methods can be used for this purpose. Nevertheless, working groups reported different results on their usage [23,24,25]. Recently, Mahajan et al. highlighted hypocomplementemia, class III or IV LN, high chronicity index, hypertension, older age, male sex, and black race as the factors that are most commonly associated with ESRD [3]. Of note, data on the white population of Central Europe is limited, and Hungarian data on the clinical characteristics of lupus nephritis are not available from the past 10 years. It is not known whether there is a difference in the course of lupus nephritis between the histological classes. It is also unknown which further factors may affect the progression of CKD. Furthermore, the differences in clinical course and laboratory parameters between patients with complete remission (CR), partial remission (PR) and no remission (NR) are not fully known. In view of all this, we found it worthwhile to investigate the parameters mentioned above and to assess prognostic factors of an unfavourable renal outcome.

## 2. Materials and Methods

### 2.1. Study Pulation

**Figure 1:**
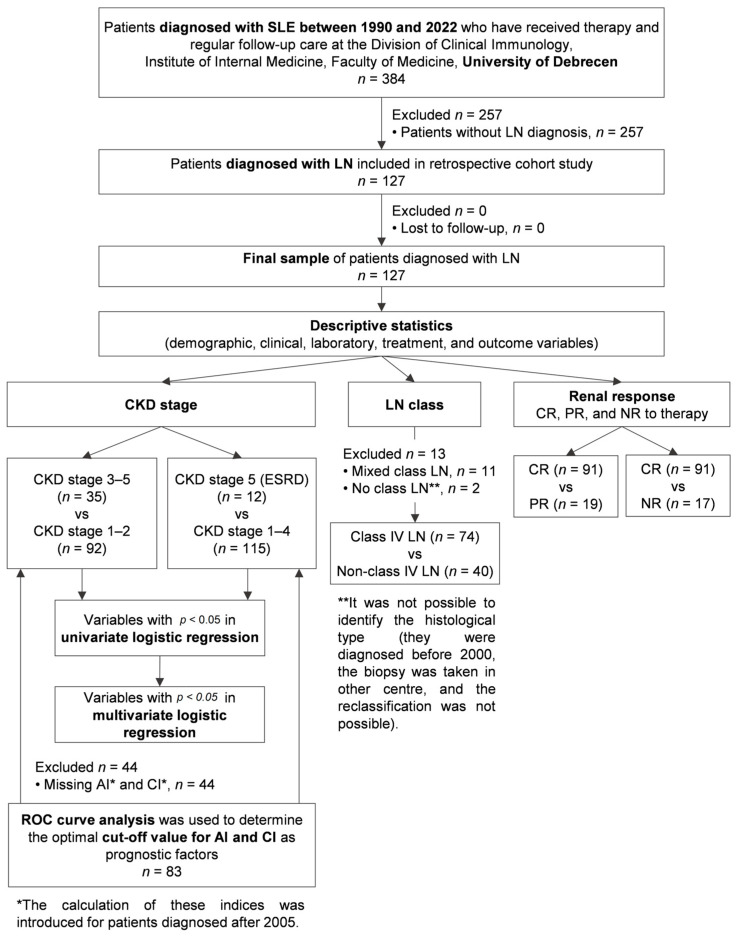
Study design flow diagram. Abbreviations: SLE, systemic lupus erythematosus; n, number of patients; LN, lupus nephritis; CKD, chronic kidney disease; ESRD, end-stage renal disease; CR, complete remission; PR, partial remission; NRs, no responders; ROC, receiver operating characteristic; AI, activity index; CI, chronicity index.

In our retrospective cohort study, we gathered the data of 384 Hungarian SLE patients, all of whom were white Europeans from the Central European region. They were diagnosed with SLE between 1990 and 2022 and have received therapy and regular follow-up care at the Division of Clinical Immunology, Institute of Internal Medicine, Faculty of Medicine, University of Debrecen. Patients who were diagnosed with SLE before 2012 were revised according to the SLICC criteria for SLE [26]; additionally, all SLE patients fulfilled the EULAR/ACR 2019 classification criteria for lupus [27]. SLE patients with secondary antiphospholipid syndrome (APS) fulfilled the 2006 Sydney criteria; of note, all of them met the criteria of the 2023 APS ACR/EULAR classification criteria, as well [28,29]. Lupus nephritis was diagnosed in 127 of them; therefore, only these patients were included in the further examinations of the present study. The clinical and laboratory data of the patients were extracted from medical documentations and records for statistical analyses. The date of data collection was January, 2024. Figure 1 shows the flow chart of this study.

### 2.2. Clinical and Laboratory Evaluation

All patients were routinely followed up throughout the studied period, and their medical records contained detailed information on medical history, treatments, clinical symptoms, physical conditions, and laboratory and other findings of each visit. The following demographic and clinical data were analysed: sex, age, age at diagnosis, disease duration, histological class of lupus nephritis, clinical symptoms and laboratory results, immunoserological abnormalities, applied treatments, complete and partial remission, and non-responsive status. We defined CKD stages according the KDIGO guidelines [30]. We combined CKD stage 1 and stage 2 into a single category. CKD stage 3 was described as eGFR 30 to <60 mL/min per 1.73 m^2^, and CKD stage 4 was described as eGFR 15 to <30 mL/min per 1.73 m^2^ for at least 3 consecutive months. CKD stage 5 (ESRD) was defined as eGFR ≤ 15 mL/min per 1.73 m^2^ for at least 3 consecutive months or ongoing dialysis. Histological sampling was carried out at the Department of Nephrology, Institute of Internal Medicine, Faculty of Medicine, University of Debrecen, and the evaluation of the kidney biopsy samples was performed at the Department of Pathology, Faculty of Medicine, University of Debrecen. We used the relevant WHO, ISN/RPS2003 and ISN/RPS2018 classification systems for the histological evaluation [9,10,11]. The calculation of activity and chronicity indices was introduced for patients who were diagnosed after 2005, meaning that 83 patients (20 with CKD stage 3–5 and 63 with CKD stage 1–2) had data about the LN Activity and Chronicity Index. Complete or partial remission of LN was defined according to the KDIGO guidelines [16]. In the case of a CR, the degree of proteinuria decreased below 0.5 g in 6 months, and the creatinine level returned to the initial value. In case of PR, the degree of proteinuria was halved in 6 months, and the serum creatinine level stabilized or improved but did not return to baseline. Chronic organ damage in SLE was determined using the SLICC damage index [31]. Immune serological parameters were determined from serum samples. The presence of antinuclear antibodies (ANAs) was detected by an indirect immunofluorescence method on the HEp2 cell line. Enzyme-linked immunosorbent assay (ELISA) was used for the detection of the following antibodies: anti-dsDNA (Orgentec, Mainz, Germany), anti-SS-A, anti-SS-B, anti-RNP, and anti-Sm (Hycor, Biomedical, Garden Grove, CA, USA), as well as antiphospholipid antibodies including anti-cardiolipin (aCL) IgG/IgA/IgM and anti-ß2GPI IgG/IgA/IgM antibodies (Orgentec, Mainz, Germany). All laboratory tests were performed under standardized conditions, according to the manufacturer’s instructions, at the Department of Laboratory Medicine, Faculty of Medicine, University of Debrecen.

### 2.3. Statistical Analysis

Statistical analysis was performed using SPSS Statistics for Windows, Version 28.0 (IBM Corporation, Armonk, NY, USA), and GraphPad Prism version 9.5 for Windows (GraphPad Software, San Diego, CA, USA). A Shapiro–Wilk test of normality was conducted to determine whether the continuous data were normally distributed. Values are expressed as median with an interquartile range (IQR) for continuous variables and frequency with percentage for categorical variables. Continuous variables were compared with a nonparametric Mann–Whitney U test. Categorical variables were compared with Pearson’s chi-squared test or Fisher’s exact test. Univariate and multivariate binary logistic regression analyses were performed to identify predictors of the progression of CKD. First, we performed a univariate analysis using variables with *p* < 0.05 in a Mann–Whitney U test or Pearson’s chi-squared test and Fisher’s exact test to avoid over-fitting; only variables with *p* < 0.05 in the univariate binary logistic regression analysis were included in the multivariate analysis. A receiver operating characteristic (ROC) curve analysis was used to determine the optimal cut-off value for AI and CI as prognostic factors of the progression of CKD into stage 3–5 and the development of ESRD. All statistical tests were two-sided, and differences were considered statistically significant at <0.05 level and reported using *p*-values and/or 95% confidence intervals (95% CIs).

## 3. Results

### 3.1. Main Analyses

The population of our retrospective study consisted of 127 LN patients, 114 (89.8%) women and 13 (10.2%) men. Their age at the time of SLE diagnosis was 28.4 ± 9.9 years, and they were followed up regularly at our autoimmune centre for 16.9 ± 8.1 years. All patients who were included in the study underwent a renal biopsy. The most common histological type was class IV LN, followed by class V and then class III. Mixed histological types were found in 11 patients, while for two patients, it was not possible to identify the histological type (they were diagnosed before 2000, the biopsy was taken in another centre, and reclassification was not possible). We did not detect class VI LN in our patient group. CKD stage 3–5 developed in 35 patients; among them, 12 patients had CKD stage 5 (ESRD). At the time of the data collection, 71.6% of patients were in CR, 15% were in PR, while 13.4% were non-responders to the therapy (Table 1).

### 3.2. Comparison of Patients Based on the Stage of CKD

We compared the demographics, clinical characteristics, laboratory parameters, therapies, outcome, and mortality of CKD stage 3–5 patients with the ones with CKD stage 1–2, as well as CKD stage 5 (ESRD) patients with the ones with CKD stage 1–4 (non-ESRD). Our findings are shown in Table 2 and Table 3.

CKD stage 3–5 patients were significantly older at the time of data collection than CKD stage 1–2 patients, while there was no difference between the ages of ESRD and non-ESRD patients. We found no differences between CKD stage 3–5 and CKD stage 1–2, nor between the ESRD and non-ESRD groups in terms of SLE clinical symptoms, autoantibodies, and cardiovascular complications. The association with Sjögren’s syndrome was significantly more common in the CKD stage 3–5 group compared to CKD stage 1–2 patients. Coombs positivity was significantly more common in CKD stage 3–5 patients, while anti-DNA positivity was significantly less common. Anti-RNP and anti-SS-B autoantibody positivity were detected significantly more frequently in ESRD patients compared to non-ESRD patients. The occurrence of class IV LN was significantly more frequent in the CKD stage 3–5 group; on the other hand, class III LN did not occur in patients with CKD stage 3–5 at all. The chronicity index, as well as SDI score, was significantly higher in both the CKD and ESRD groups compared to CKD stage 1–2 and non-ESDR patients. The administration of immunosuppressive therapy did not differ between CKD stage 3–5 and CKD stage 1–2 or between the ESRD and non-ESRD groups. The cumulative steroid dose was significantly higher in the CKD stage 3–5 group compared to patients with CKD stage 1–2. A CR developed significantly less frequently in CKD stage 3–5, as well as in the ESRD group. None of the ESRD patients reached CR. Consequently, the ratio of non-responder patients was significantly higher in both the CKD and ESRD groups. The total mortality rate was significantly higher in patients with CKD stage 3–5 than in patients with CKD stage 1–2; however, there was no difference regarding the cause of death.

### 3.3. Prognostic Factors for the Progression of CKD and the Development of ESRD

Univariate and multivariate logistic regression analyses revealed that age above 42 years, class IV lupus nephritis, Coombs positivity, and a higher chronicity index are important prognostic factors of the development of CKD stage 3–5 (Table 4).

Univariate logistic regression analysis revealed that anti-RNP and anti-SS-B positivity, as well as a high chronicity index, are important prognostic factors of the development of ESRD. Of note, the multivariate logistic regression analysis only confirmed the high chronicity index among these (Table 5).

**Figure 2:**
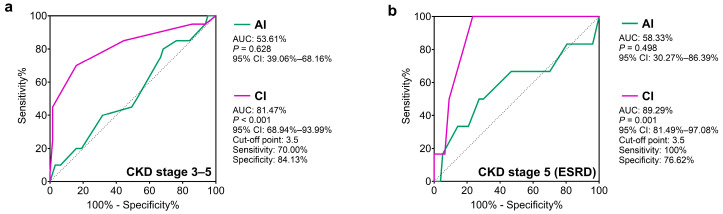
ROC curve and analysis of cut-off values of AI and CI in relation to CKD stage 3–5 (**a**) and ESRD (**b**). Total number of LN patients is 127, of which 83 patients (20 CKD stage 3–5 and 63 CKD stage 1–2; 6 ESRD and 77 non-ESRD) had data about LN Activity and Chronicity Index. Abbreviations: AI, activity index; CI, chronicity index; CKD, chronic kidney disease; ESRD, end-stage renal disease; ROC, receiver operating characteristic; AUC, area under the ROC curve; 95% CI, 95% confidence interval.

We also evaluated the possible associations between activity and chronicity index scores and the development of CKD stage 3–5 and ESRD. Figure 2 shows the results of the ROC analysis. The activity score did not show any correlation with the development of CKD stage 3–5 or ESRD. However, a chronicity index score above 3.5 points showed a significant correlation with the development of both CKD stage 3–5 and ESRD.

### 3.4. Differences Between Class IV LN and Non-Class IV LN Patients

Given that class IV LN was the most common histological type and a prognostic factor of the development of CKD stage 3–5, we compared it with other non-class IV LN patients. The results are summarized in Table 6 and Table 7.

Class IV LN patients were younger than the others at the time of data collection; however, their age at diagnosis did not differ. Regarding the clinical features, deep vein thrombosis, as well as rheumatoid arthritis, was significantly less common in class IV LN patients. During the assessment of kidney pathology, we found that both the activity and chronicity indexes were significantly higher, and the development of CKD stage 3–5 was more prevalent in the class IV lupus nephritis patients. We did not find any difference in immunoserological results. Azathioprine was significantly more frequently used as a maintenance treatment in the class IV LN group, while cyclosporine A was used significantly less often. Total mortality was not more frequent in the class IV lupus nephritis group, but among the causes of death, tumour mortality was significantly less frequent.

### 3.5. Differences Between Complete, Partial, and Non-Responders

We also assessed the clinical and laboratory parameters of the patients with CR, PR, and NR. The results are summarized in Table 8.

The age at data collection was significantly higher, and the duration of disease was significantly longer, in the patients in CR than the patients in PR. Regarding autoantibodies, no differences were found between the three groups. The occurrence of CKD stage 3–5 and ESRD was significantly more frequent among non-responders compared to patients in CR, and their chronicity index was also significantly higher. Steroid use was significantly more frequent among patients in PR compared to patients in CR. MMF induction and maintenance treatments were used significantly more often in patients in PR. MMF induction therapy was significantly more common among non-responders compared to patients in CR. Both tacrolimus and belimumab were used significantly more often in patients in PR than in CR. Patients in CR achieved lupus low-disease-activity state (LLDAS) more often than patients in PR. The SDI score was significantly higher among non-responders compared to those in CR.

## 4. Discussion

Lupus nephritis is one of the most serious organ manifestations of SLE, in addition to neuropsychiatric manifestations [32]. LN usually manifests in the first five years of the disease and may be the only clinical manifestation of SLE. This latter fact is taken into account by the latest EULAR/ACR classification criteria system, according to which, in addition to the entry criteria, the presence of histologically confirmed class IV lupus nephritis is sufficient to establish a diagnosis of SLE [27]. The most common histological type is class IV LN [3,33,34], and we found it in nearly 60% of our patients. A similar frequency was reported by Vajgel et al., although, in a non-white population [35]. CKD stage 3–5 developed in 27.6% of our patients. However, when comparing our observations with the results of other working groups, it is important to highlight the fact that LN implies persistent alterations to kidney structures and functions; therefore, all LN patients develop CKD, and the CKD stage of these patients determines their health outcomes [5]. In the publications that have been published so far in the literature, the definition used by each working group for the diagnosis of CKD in their LN patients corresponded to our definition of CKD stages 3–5 (defined by eGFR < 60 mL/min per 1.73 m^2^ for at least 3 consecutive months). Consequently, their definition for non-CKD LN patients corresponds to the definition of LN patients with CDK stage 1–2 in our study. An Egyptian working group observed a CKD rate of 32.7% in a 5-year study of more than 900 patients with lupus nephritis; however, these data come from a non-white population [36]. The frequency of ESRD in our patient population was 9.4%, which is similar to the data in the RELESSER register (10.35%) [37]. Platinga et al. found the 5-year cumulative incidence of ESRD to be 2.5% in white and 6.4% in black individuals [38]. Similar results were obtained by Hanly et al., who found that for all patients with LN, the 5-year cumulative incidence of ESRD was 3.3%, while the 10-year cumulative incidence was 4.3% [24]. When we evaluated the demographic, clinical, and laboratory data and renal outcomes of CKD stage 3–5 patients, we found that they were older, and an association with Sjögren’s syndrome was more common, compared to the patients with CKD stage 1–2. Park et al. found CKD patients to be similarly older in a Korean population. Similarly to these results, the clinical manifestations of SLE did not differ between our patients in the CKD stage 3–5 and CKD stage 1–2 groups. At the same time, in the Korean cohort, hypertension, elevated serum creatinine levels, reduced eGFR, and nephrotic proteinuria were more common in the CKD group at the time of LN diagnosis [39]. We could not examine these parameters, as the initial laboratory parameters were not available for all patients due to the long follow-up period. We found that class IV lupus nephritis and a higher chronicity index are significantly more common in patients with CKD stage 3–5. It is similar to the results of other studies; however, we found no correlation with the activity scores [40,41]. Direct Coombs positivity was also more common in the CKD stage 3–5 group, but anti-DNA positivity was significantly less frequent, which contradicts the literature data. Based on a Spanish register’s data, ESRD showed a significant association with anti-DNA positivity; however, it was also described that there is a significant correlation between hemolytic anaemia and ESRD, and they did not comment on the isolated Coombs positivity [37]. Regarding therapy, we found no difference between the CKD stage 3–5 and CKD stage 1–2 groups, except for the higher cumulative steroid dose. The reason for this may be that CKD patients had a more severe form of LN, and a higher dose of steroid was needed. Not surprisingly, among patients with CKD stage 3–5, there were fewer patients in CR and more non-responders. We observed higher SDI scores and mortality rates in the CKD stage 3–5 group, but there was no difference in causes of death. Based on our results, age above 42 years, class IV LN, Coombs positivity, and a higher chronicity index proved to be important prognostic factors of the development of CKD stage 3–5. Formerly, several working groups identified hypertension, elevated serum creatinine levels, and a lack of remission as prognostic factors [3,35]. A higher chronicity index, on the other hand, is described by numerous working groups as a prognostic factor for CKD and ESRD [3,39]. We found a score of 3.5 points to be the value above which the development of CKD stage 3–5 and ESRD must be more anticipated.

We compared the data of ESRD and non-ESRD patients as well. We found no differences in the clinical symptoms of SLE, but anti-RNP and anti-SS-B positivity were significantly more frequent in the ESRD group. Several working groups have described anti-SS-A as an independent predictor of ESRD, without mentioning anti-SS-B [42,43]. Anti-RNP and anti-SS-B positivity, as well as a higher chronicity index, were found to be prognostic factors for the development of ESRD; however, our multivariate analysis only confirmed the prognostic value of a higher chronicity index. Other study groups found hypocomplementemia, class III, IV, VI lupus nephritis, hypertension, older age, male sex, and black race to be the main prognostic factors for ESRD [35]. We did not find class III LN to be more frequent; moreover, this class did not even occur in neither the CKD stage 3–5, nor the ESRD group.

Given that class IV LN was the most common histological type, and CKD stage 3–5 and ESRD did not occur in class III LN patient group, as a next step, we compared class IV and non-class IV LN patients. Duran et al. examined the clinical characteristics and disease outcome of proliferative and non-proliferative LN patients. They found no difference in SLE clinical symptoms between the two groups, just as we found no difference between the class IV and non-class IV LN patients. They found significant differences, however, in several parameters indicating kidney activity, such as the serum creatinine level, eGFR, proteinuria, hypocomplementemia, active urine sedimentation, and renal SLE DAI [44]. We did not examine these parameters in our study. We found a significantly higher activity and chronicity index in the class IV LN patients. A Columbian working group obtained similar results when comparing patients with proliferative and non-proliferative LN [45]. In terms of therapy, we found a difference only in the administration of cyclosporine A. We found that this was used significantly less often in class IV LN, similarly to Duran et al. [44]. We found no difference in mortality between patients with class IV and non-class IV LN; however, the tumour mortality was significantly lower among class IV LN patients.

Finally, we also compared patients who were in CR or PR and non-responders. Patients in CR were older and had a longer disease duration compared to patients in PR. The damage index and the prevalence of CKD stage 3–5 and ESRD were significantly higher among the non-responders. We found no differences between the individual groups in terms of clinical symptoms and laboratory abnormalities, but there was a difference in their treatment. Patients in PR received newer drugs, such as MMF, tacrolimus, and belimumab significantly more frequently. In the therapeutic recommendations for lupus nephritis, these drugs have been introduced in recent years, and the latest KDIGO guidelines and ACR/EULAR recommendations also include them [15,16]. Presumably, the newer and registered drugs will be used more and more widely, not only in patients in PR, but also in the entire lupus nephritis patient population. Among immunosuppressive treatments, steroid therapy was also more common in patients in PR. Patients in CR took steroids less often, which is also in line with the current therapeutic guidelines and our therapeutic efforts. Nevertheless, even more attention should be paid to the use of antimalarial drugs, because half of our patients only received antimalarial drugs, even though it is well known that HCQ has a beneficial effect on the outcome of the disease in many respects. Park et al. also reported that the use of hydroxychloroquine before the onset of lupus nephritis protects patients from developing CKD [39]. When examining the disease outcome, we found that mortality did not differ between the groups, but LLDA was significantly more prevalent in the CR group, which may improve the morbidity and mortality indicators of patients in the long term. At the same time, we found a higher damage index among non-responders, which may have a negative effect on the long-term outcome.

This study has some limitations. It was a retrospective study, and some risk factors could not be included in the analysis as covariates or potential confounders. Regarding the treatment adherence of the included patients, despite knowing the medications, the doses used, and the high level of accuracy of the administrative staff based on the documentation of drug prescriptions, we cannot exclude the possibility of erratic patient adherence. Despite these limitations, our study had two major strengths. One of them is the length of the period examined, which was more than three decades in some cases (mean follow-up time was 16.9 ± 8.1 years). Another strength is that we investigated and analysed multiple clinical parameters and disease outcomes, providing more real-world data about the natural course of LN. Nevertheless, our observations have to be confirmed in a larger patient population as well; moreover, further studies are needed to elucidate the pathophysiological role of Coombs positivity and anti-RNP and anti-SS-B antibodies in the progression of LN.

In conclusion, class IV lupus nephritis is the most common form of LN in our centre, in which the renal outcome is clearly unfavourable. Age above 42 and Coombs positivity are also prognostic for the development of CKD stage 3–5, while a high chronicity index is prognostic for both CKD stage 3–5 and ESRD. Based on our results, the development of CKD stage 3–5 or ESRD should be expected at a chronicity index above 3.5 points. The chronicity index, as well as SLICC/ACR Damage Index (SDI) score, was significantly higher in non-responders compared to patients with complete remission. Among patients in CR, we achieved LLDA more often, and the average damage score and steroid use were significantly lower. Hopefully, the newly registered drugs and the ones that are still under development will enable a higher rate of CR in lupus nephritis patients, which will have a favourable effect on the long-term outcome.

## Figures and Tables

**Table 1:** Characteristics of LN patients (*n* = 127).

	*n*	%
*Kidney biopsy*	127	100
ISN/RPS classification		
Class I-II	7	5.5
Class III	13	10.2
Class IV	74	58.3
Class V	20	15.7
Class VI	0	0.0
Mixed class ^a^	11	8.7
Not classified	2	1.6
*Renal complications*		
CKD stage 1–2	92	72.4
CKD stage 3	8	6.3
CKD stage 4	15	11.8
CKD stage 5 (ESRD)	12	9.4
*Renal response*		
CR	91	71.6
PR	19	15.0
NR	17	13.4

Values are presented as number and percentage (%). ^a^ Mixed LN class: Class I-II + III: 1 patient; Class I-II + IV: 1 patient; Class I-II + V: 1 patient; Class III + IV: 2 patients; Class III + V: 5 patients; Class IV + V: 1 patient. Abbreviations: LN, lupus nephritis; *n*, number of patients; ISN/RPS, International Society of Pathology/Renal Pathology Society; CKD, chronic kidney disease; ESRD, end-stage renal disease; CR, complete remission; PR, partial remission; NR, no remission.

**Table 2:** Comparison of demographics, clinical features, and positive laboratory findings between the groups of LN patients with different CKD stages (*n* = 127).

	CKD Stage 3–5 (*n* = 35)	CKD Stage 1–2 (*n* = 92)	*p*-Value	CKD Stage 5 (ESRD) (*n* = 12)	CKD Stage 1–4 (Non-ESRD) (*n* = 115)	*p*-Value
**Demographics**						
Sex (female)	30 (85.7)	84 (91.3)	0.345	12 (100)	102 (88.7)	0.611
Age, years	49 (44–54)	43 (36–50)	0.016 *	49 (43.5–51.5)	45 (37–53)	0.465
Age at onset of SLE, years	31 (21.5–39)	26 (20.5–34)	0.067	23 (20–24)	27 (21–35)	0.428
Duration of SLE, years	18 (11.5–24)	16 (10.5–22)	0.390	23 (14–26.5)	16 (11–22)	0.124
**Extrarenal clinical features**						
*Mucocutan*						
Vasculitis	6 (17.1)	24 (26.1)	0.289	4 (33.3)	26 (22.6)	0.476
Livedo reticularis	3 (8.6)	8 (8.7)	1.000	1 (8.3)	10 (8.7)	1.000
Acute skin lesions	12 (34.3)	41 (44.6)	0.294	4 (33.3)	49 (42.6)	0.535
DLE	3 (8.6)	3 (3.3)	0.345	2 (16.7)	4 (3.5)	0.099
SCLE	3 (8.6)	4 (4.3)	0.393	1 (8.3)	6 (5.2)	0.510
Alopecia	14 (40.0)	24 (26.1)	0.126	5 (41.7)	33 (28.7)	0.342
Photosensitivity	8 (22.9)	21 (22.8)	0.997	4 (33.3)	25 (21.7)	0.468
Mucous ulcer	6 (17.1)	10 (10.9)	0.375	2 (16.7)	14 (12.2)	0.648
*Serositis*						
Pleuritis	9 (25.7)	23 (25)	0.934	5 (41.7)	27 (23.5)	0.176
Pericarditis	9 (25.7)	16 (17.4)	0.292	4 (33.3)	21 (18.3)	0.251
*Neuropsychiatric*						
CNS manifest.	6 (17.1)	19 (20.7)	0.657	2 (16.7)	23 (20.0)	1.000
PNS manifest.	4 (11.4)	6 (6.5)	0.461	0 (0)	10 (8.7)	0.596
Psychiatric manifest.	6 (17.1)	19 (20.7)	0.657	2 (16.7)	23 (20.0)	1.000
*Cardiovascular*						
APS	6 (17.1)	15 (16.3)	0.910	2 (16.7)	19 (16.5)	1.000
DVT	3 (8.6)	14 (15.2)	0.396	0 (0)	17 (14.8)	0.366
PE	1 (2.9)	3 (3.3)	1.000	1 (8.3)	3 (2.6)	0.331
AMI	1 (2.9)	2 (2.2)	1.000	1 (8.3)	2 (1.7)	0.259
Stroke	1 (2.9)	7 (7.6)	0.443	1 (8.3)	7 (6.1)	0.559
Valvulopathy	9 (25.7)	16 (17.4)	0.292	3 (25.0)	22 (19.1)	0.703
*Others*						
Polyarthritis	31 (88.6)	80 (87.0)	1.000	10 (83.3)	101 (87.8)	0.648
SpAb^a^	5 (16.7)	15 (17.9)	0.883	2 (16.7)	18 (17.6)	1.000
*Associated autoimmune diseases*						
RA	3 (8.6)	10 (10.9)	1.000	1 (8.3)	12 (10.4)	1.000
SS	5 (14.3)	0 (0)	0.001 *	1 (8.3)	4 (3.5)	0.396
PSS	0 (0)	2 (2.2)	1.000	0 (0)	2 (1.7)	1.000
**Laboratory**						
Thrombocytopenia	17 (48.6)	41 (44.6)	0.685	5 (41.7)	53 (46.1)	0.770
Leukopaenia	27 (77.1)	70 (76.1)	0.900	10 (83.3)	87 (75.7)	0.730
Anaemia	29 (82.9)	81 (88.0)	0.560	9 (75)	101 (87.8)	0.203
Anti-ß2-GP-1	21 (60)	46 (50.0)	0.313	7 (58.3)	60 (52.2)	0.684
Anti-CL	25 (71.4)	58 (63.0)	0.375	8 (66.7)	75 (65.2)	1.000
LA	7 (20)	16 (17.4)	0.733	3 (25.0)	20 (17.4)	0.454
Anti-dsDNA	30 (85.7)	90 (97.8)	**0.017 ^#^**	11 (91.7)	109 (94.8)	0.510
Anti-Sm	18 (51.4)	34 (37.0)	0.138	6 (50.0)	46 (40.0)	0.548
Anti-RNP	17 (48.6)	29 (31.5)	0.074	8 (66.7)	38 (33.0)	0.029 *
Anti-SS-A (Ro)	20 (57.1)	57 (62.0)	0.620	9 (75.0)	68 (59.1)	0.362
Anti-SS-B (La)	18 (51.4)	33 (35.9)	0.110	9 (75.0)	42 (36.5)	0.013 *
ANCA	5 (14.3)	12 (13.0)	1.000	2 (16.7)	15 (13.0)	0.663
Cryoglobulin	2 (5.7)	5 (5.4)	1.000	0 (0)	7 (6.1)	1.000
Coombs test (+)	10 (28.6)	10 (10.9)	0.014 *	4 (33.3)	16 (13.9)	0.096

Values are presented as frequency (%) or median (IQR), while *p*-values are calculated by Pearson’s chi-squared test or Fisher’s exact test and Mann–Whitney U test. Statistically significant variables (*p* < 0.05) are highlighted as bold text. ^a^ There are 114 women in the group. * Significantly more common in CKD stage 3–5 or ESRD group. **^#^** Significantly less common in CKD stage 3–5 or ESRD group. Abbreviations: LN, lupus nephritis; n, number of patients; CKD, chronic kidney disease; ESRD, end-stage renal disease; DLE, disocid lupus erythematosus; SCLE, subacute cutaneous lupus; CNS manifest., central nervous system manifestations; PNS manifest, peripheral nervous system manifestations; APS, antiphospholipid syndrome; DVT, deep vein thrombosis; PE, pulmonary embolism; AMI, acute myocardial infarction; SpAB, spontaneous abortion; RA, rheumatoid arthritis; SS, Sjögren’s syndrome; PSS, progressive systemic sclerosis; anti-ß2GPI, anti-β2-glycoprotein-1 antibody; anti-CL, anticardiolipin antibody; LA, lupus anticoagulant; anti-dsDNA, anti-double-stranded DNA; anti-Sm, anti-Smith antibody; anti-RNP, anti-ribonucleoprotein antibody; anti-SS-A, anti-Sjögren’s-syndrome-related antigen A autoantibody; anti-SS-B, anti-Sjögren’s-syndrome-related antigen B autoantibody; ANCA, antineutrophil cytoplasmic antibody; n.c., not computable.

**Table 3:** Comparison of renal pathological findings, treatment, renal response, and outcomes between the groups of LN patients with different CKD stages (*n* = 127).

	CKD Stage 3–5 (*n* = 35)	CKD Stage 1–2 (*n* = 92)	*p*-Value	CKD Stage 5 (ESRD) (*n* = 12)	CKD Stage 1–4 (Non-ESRD) (*n* = 115)	*p*-Value
**Renal pathological findings**						
ISN/RPS LN Class						
I-II	2 (5.7)	5 (5.4)	1.000	0 (0)	7 (6.1)	1.000
III	0 (0)	13 (14.1)	**0.019 ^#^**	0 (0)	13 (11.3)	0.611
IV	26 (74.3)	48 (52.2)	0.024 *	9 (75.0)	65 (56.5)	0.217
V	4 (11.4)	16 (17.4)	0.410	2 (16.7)	18 (15.7)	1.000
Mixed Class LN ^a^	2 (5.7)	9 (9.8)	0.726	1 (8.3)	10 (8.7)	1.000
No Class LN ^a^	1 (2.9)	1 (1.1)	0.477	0 (0)	2 (1.7)	1.000
AI (*n* = 83), score ^b^	9.5 (6–10.5)	9 (6-12)	0.626	11 (6–15)	9 (6–12)	0.496
CI (*n* = 83), score ^b^	4 (3–5.5)	2 (2-3)	<0.001 *	4.5 (4-5)	3 (2-3)	0.001 *
**Treatment**						
Steroid (currently)	23 (65.7)	58 (63.0)	0.780	7 (58.3)	74 (64.3)	0.756
Chloroquine	14 (40.0)	46 (50.0)	0.313	6 (50.0)	54 (47.0)	0.841
AZA	25 (71.4)	71 (77.2)	0.501	9 (75.0)	87 (75.7)	1.000
Maintenance Th	19 (54.3)	62 (67.4)	0.170	7 (58.3)	74 (64.3)	0.756
MMF						
Induction Th	18 (51.4)	34 (37.0)	0.138	6 (50.0)	46 (40.0)	0.548
Maintenance Th	9 (25.7)	26 (28.3)	0.774	2 (16.7)	33 (28.7)	0.509
CYC	32 (91.4)	71 (77.2)	0.067	12 (100)	91 (79.1)	0.121
MTX	5 (14.3)	7 (7.6)	0.309	0 (0)	12 (10.4)	0.603
CsA	2 (5.7)	11 (12)	0.513	0 (0)	13 (11.3)	0.611
Tacrolimus	4 (11.4)	8 (8.7)	0.736	3 (25.0)	9 (7.8)	0.087
Plasmapheresis	8 (22.9)	18 (19.6)	0.681	5 (41.7)	21 (18.3)	0.069
Rituximab	6 (17.1)	12 (13.0)	0.575	1 (8.3)	17 (14.8)	1.000
Belimumab	1 (2.9)	7 (7.6)	0.443	0 (0)	8 (7.0)	1.000
CD, mg/kg	23,360 (11,990–36,500)	14,600 (5840–29,200)	0.048 *	22,630 (13,870–37,960)	14,600 (7300–32,120)	0.362
**Renal response**						
CR	16 (45.7)	75 (81.5)	**<0.001 ^#^**	0 (0)	91 (79.1)	**<0.001 ^#^**
PR	4 (11.4)	15 (16.3)	0.491	1 (8.3)	18 (15.7)	0.692
NR	15 (42.9)	2 (2.2)	<0.001 *	11 (91.7)	6 (5.2)	<0.001 *
**Outcomes**						
SDI, score	2 (1–3)	0 (0–1)	<0.001 *	3 (1.5–3.5)	0 (0–1)	<0.001 *
LLDAS	8 (22.9)	15 (16.3)	0.392	3 (25.0)	20 (17.4)	0.454
Remission	4 (11.4)	18 (19.6)	0.279	2 (16.7)	20 (17.4)	1.000
Mortality	9 (25.7)	9 (9.8)	0.042 *	2 (16.7)	16 (13.9)	0.679
Cardiovascular	3 (33.3)	2 (22.2)	1.000	0 (0)	5 (31.3)	1.000
Infection	1 (11.1)	2 (22.2)	1.000	1 (50.0)	2 (12.5)	0.314
Sepsis	4 (44.4)	1 (11.1)	0.294	1 (50.0)	4 (25.0)	0.490
Tumour	0 (0)	3 (33.3)	0.206	0 (0)	3 (18.8)	1.000
Underlying disease	1 (11.1)	1 (11.1)	1.000	0 (0)	2 (12.5)	1.000

Values are presented as frequency (%) or median (IQR), while *p*-values are calculated by Pearson’s chi-squared test or Fisher’s exact test and Mann–Whitney U test. Statistically significant variables (*p* < 0.05) are highlighted as bold text. ^a^ Total number of LN patients is 127, of which 11 patients had Mixed LN Class (Class I-II + III: 1 patient; Class I-II + IV: 1 patient; Class I-II + V:1 patient; Class III + IV: 2 patients; Class III + V: 5 patients; Class IV + V: 1 patient), and 2 patients had Not Classified LN. ^b^ The total number of LN patients is 127, of which 83 patients had data about LN Activity and Chronicity Index. The calculation of these indices was introduced for patients who were diagnosed after 2005. * Significantly more common in CKD stage 3–5 or ESRD group. **^#^** Significantly less common in CKD stage 3–5 or ESRD group. Abbreviations: LN, lupus nephritis; *n*, number of patients; CKD, chronic kidney disease; ESRD, end-stage renal disease; ISN/RPS, International Society of Pathology/Renal Pathology Society; AI, activity index; CI, chronicity index; AZA, azathioprine; MMF, mycophenolate mofetil; Th, therapy; CYC, cyclophosphamide; MTX, methotrexate; CsA, cyclosporine A; CD, cumulative dose; CR, complete remission; PR, partial remission; NR, no remission; SDI, SLICC/ACR Damage Index, LLDAS, lupus low-disease-activity state; n.c., not computable.

**Table 4:** Univariate and multivariate logistic regression analyses of the prognostic factors of the development of CKD stage 3–5 (*n* = 35) in patients with LN (*n* = 127).

	Univariate Analysis		Multivariate Analysis	
Variables	OR (95% CI)	*p*-Value	OR (95% CI)	*p*-Value
Age ≥ 42 years	5.70 (1.86–17.49)	**0.002**	13.12 (1.55–111.36)	**0.018**
Class IV LN	2.65 (1.12–6.27)	**0.027**	7.61 (1.13–51.01)	**0.037**
Anti-dsDNA	0.13 (0.02–0.72)	**0.020**	0.15 (0.01–1.87)	0.141
Anti-RNP	2.05 (0.93–4.55)	0.077		
Anti-SS-B (La)	1.89 (0.86–4.16)	0.112		
Coombs test positivity	3.28 (1.23–8.78)	**0.018**	17.2 (1.98–149.51)	**0.010**
AI, score *	0.96 (0.86–1.07)	0.481		
CI, score *	2.65 (1.60–4.38)	**<0.001**	2.14 (1.3–3.52)	**0.003**

Values are presented as odds ratio (95% CI), while *p*-values are calculated by univariate and multivariate logistic regression analysis. Statistically significant variables (*p* < 0.05) are highlighted as bold text. * Total number of LN patients is 127, of which 83 patients (20 patients with CKD stage 3–5 and 63 patients with CKD stage 1–2) had data about LN Activity and Chronicity Index. The calculation of these indices was introduced for patients who were diagnosed after 2005. Abbreviations; LN, lupus nephritis; CKD, chronic kidney disease; OR, odds ratio; 95% CI, 95% confidence interval; anti-dsDNA, anti-double-stranded DNA; anti-RNP, anti-ribonucleoprotein antibody; anti-SS-B, anti-Sjögren’s-syndrome-related antigen B autoantibody; AI, activity index; CI, chronicity index.

**Table 5:** Univariate and multivariate logistic regression analyses of the prognostic factors of the development of CKD stage 5 (ESRD) (*n* = 12) in patients with LN (*n* = 127).

	Univariate Analysis		Multivariate Analysis	
Variables	OR (95% CI)	*p*-Value	OR (95% CI)	*p*-Value
Age ≥ 42 years	2.77 (0.58–13.25)	0.202		
Class IV LN	2.31 (0.59–8.97)	0.227		
Anti-dsDNA	0.61 (0.07–5.50)	0.656		
Anti-RNP	4.05 (1.15–14.31)	**0.030**	3.00 (0.28–32.27)	0.364
Anti-SS-B (La)	5.21 (1.34–20.33)	**0.017**	1.20 (0.12–12.02)	0.876
Coombs test positivity	3.09 (0.83–11.48)	0.091		
AI, score *	1.04 (0.87–1.25)	0.650		
CI, score *	2.37 (1.27–4.44)	**0.007**	2.50 (1.15–5.43)	**0.021**

Values are presented as odds ratio (95% CI), and *p*-values are calculated by univariate and multivariate logistic regression analysis. Statistically significant variables (*p* < 0.05) are highlighted as bold text. * Total number of LN patients is 127, of which 83 patients (6 ESRD and 77 non-ESRD) had data about LN Activity and Chronicity Index. The calculation of these indices was introduced for patients who were diagnosed after 2005. Abbreviations; LN, lupus nephritis; ESRD, end-stage renal disease; OR, odds ratio; 95% CI, 95% confidence interval; anti-dsDNA, anti-double-stranded DNA; anti-RNP, anti-ribonucleoprotein antibody; anti-SS-B, anti-Sjögren’s-syndrome-related antigen B autoantibody; AI, activity index; CI, chronicity index.

**Table 6:** Comparison of demographics, extrarenal clinical features, and positive laboratory findings of ISN/RPS Class IV LN patients with other classes of LN patients.

	All Classes (*n* = 114) ^a^	Class I-II (*n* = 7)	Class III (*n* = 13)	Class V (*n* = 20)	Class IV (*n* = 74)	*p*-Value ^b^
***Demographics***						
Sex (female)	101 (88.6)	7 (100)	12 (92.3)	19 (95.0)	63 (85.1)	0.135
Age, years	45 (37–52)	47 (45.5–60.5)	45 (42–58)	48.5 (40.5–53)	43.5 (35–51)	0.044 *
Age at onset of SLE, years	26 (21–35)	38 (19–40)	28 (19–35)	28 (23–34.5)	25.5 (20–33)	0.231
Duration of SLE, years	16 (11–24)	27 (15–29.5)	18 (12–23)	17 (10–26.5)	16 (11–22)	0.190
***Extrarenal clinical features***						
*Mucocutan*						
Livedo reticularis	11 (9.6)	1 (14.3)	0 (0)	3 (15.0)	7 (9.5)	1.000
Acute skin lesions	45 (39.5)	2 (28.6)	5 (38.5)	6 (30.0)	32 (43.2)	0.263
DLE	6 (5.3)	0 (0)	0 (0)	0 (0)	6 (8.1)	0.089
SCLE	6 (5.3)	0 (0)	0 (0)	1 (5.0)	5 (6.8)	0.663
Alopecia	36 (31.6)	2 (28.6)	3 (23.1)	10 (50.0)	21 (28.4)	0.317
Photosensitivity	25 (21.9)	1 (14.3)	5 (38.5)	5 (25.0)	14 (18.9)	0.291
Mucous ulcer	14 (12.3)	0 (0)	2 (15.4)	1 (5.0)	11 (14.9)	0.372
*Serositis*						
Pleuritis	29 (25.4)	1 (14.3)	2 (15.4)	6 (30.0)	20 (27)	0.596
Pericarditis	23 (20.2)	1 (14.3)	3 (23.1)	3 (15.0)	16 (21.6)	0.601
*Neuropsychiatric*						
CNS manifestations	22 (19.3)	0 (0)	3 (23.1)	4 (20.0)	15 (20.3)	0.721
PNS manifestations	9 (7.9)	1 (14.3)	1 (7.7)	2 (10.0)	5 (6.8)	0.718
Psychiatric manifestations	24 (21.1)	1 (14.3)	3 (23.1)	6 (30.0)	14 (18.9)	0.447
*Cardiovascular*						
APS	18 (15.8)	1 (14.3)	4 (30.8)	2 (10.0)	11 (14.9)	0.713
DVT	14 (12.3)	1 (14.3)	4 (30.8)	4 (20.0)	5 (6.8)	**0.033 ^#^**
PE	3 (2.6)	0 (0)	0 (0)	0 (0)	3 (4.1)	0.551
AMI	3 (2.6)	0 (0)	0 (0)	1 (5.0)	2 (2.7)	1.000
Stroke	8 (7.0)	0 (0)	1 (7.7)	1 (5.0)	6 (8.1)	0.711
Vasculitis	30 (26.3)	1 (14.3)	3 (23.1)	6 (30.0)	20 (27)	0.815
Valvulopathy	23 (20.2)	2 (28.6)	1 (7.7)	5 (25.0)	15 (20.3)	0.973
*Others*						
Polyarthritis	100 (87.7)	7 (100)	10 (76.9)	18 (90.0)	65 (87.8)	1.000
SpAb^c^	18 (17.8)	2 (28.6)	4 (33.3)	3 (15.8)	9 (14.3)	0.232
*Associated autoimmune diseases*						
RA	12 (10.5)	1 (14.3)	2 (15.4)	5 (25.0)	4 (5.4)	**0.024 ^#^**
SS	4 (3.5)	0 (0)	0 (0)	1 (5.0)	3 (4.1)	1.000
PSS	2 (1.8)	0 (0)	2 (15.4)	0 (0)	0 (0)	0.121
***Laboratory***						
Thrombocytopenia	54 (47.4)	3 (42.9)	8 (61.5)	6 (30.0)	37 (50)	0.444
Leukopaenia	87 (76.3)	4 (57.1)	11 (84.6)	12 (60.0)	60 (81.1)	0.104
Anaemia	98 (86.0)	6 (85.7)	10 (76.9)	18 (90.0)	64 (86.5)	0.827
Anti-ß2-GP-1	57 (50.0)	4 (57.1)	9 (69.2)	10 (50.0)	34 (45.9)	0.239
Anti-CL	71 (62.3)	6 (85.7)	10 (76.9)	13 (65.0)	42 (56.8)	0.098
LA	20 (17.5)	1 (14.3)	3 (23.1)	3 (15.0)	13 (17.6)	0.993
Anti-dsDNA	109 (95.6)	7 (100)	12 (92.3)	19 (95.0)	71 (95.9)	1.000
Anti-Sm	48 (42.1)	4 (57.1)	3 (23.1)	10 (50.0)	31 (41.9)	0.950
Anti-RNP	41 (36)	3 (42.9)	2 (15.4)	12 (60.0)	24 (32.4)	0.285
Anti-SS-A (Ro)	69 (60.5)	5 (71.4)	8 (61.5)	16 (80.0)	40 (54.1)	0.054
Anti-SS-B (La)	45 (39.5)	4 (57.1)	3 (23.1)	11 (55.0)	27 (36.5)	0.375
ANCA	16 (14.0)	2 (28.6)	1 (7.7)	1 (5.0)	12 (16.2)	0.362
Cryoglobulin	6 (5.3)	0 (0)	1 (7.7)	0 (0)	5 (6.8)	0.663
Coombs test positivity	17 (14.9)	1 (14.3)	3 (23.1)	1 (5.0)	12 (16.2)	0.595

Values are presented as frequency (%) or median (IQR), while *p*-values are calculated by Pearson’s chi-squared test or Fisher’s exact test and Mann–Whitney U test. Statistically significant variables (*p* < 0.05) are highlighted as bold text. ^a^ Total number of LN patients is 127, of which 11 patients had Mixed LN Class (Class I-II + III: 1 patient; Class I-II + IV: 1 patient; Class I-II + V:1 patient; Class III + IV: 2 patients; Class III + V: 5 patients; Class IV + V: 1 patient), and 2 patients had Not Classified LN. These two groups were not analysed in this case. ^b^ Class IV vs. I-II+III+V Classes together. ^c^ There are 101 women in the group. * Significantly higher in Class IV. **^#^** Significantly lower in Class IV. Abbreviations: LN, lupus nephritis; n, number of patients; DLE, disocid lupus erythematosus; SCLE, subacute cutaneous lupus; CNS manifest., central nervous system manifestations; PNS manifest, peripheral nervous system manifestations; APS, antiphospholipid syndrome; DVT, deep vein thrombosis; PE, pulmonary embolism; AMI, acute myocardial infarction; SpAB, spontaneous abortion; RA, rheumatoid arthritis; SS, Sjögren’s syndrome; PSS, progressive systemic sclerosis; anti-ß2GPI, anti-β2-glycoprotein-1 antibody; anti-CL, anticardiolipin antibody; LA, lupus anticoagulant; anti-dsDNA, anti-double-stranded DNA; anti-Sm, anti-Smith antibody; anti-RNP, anti-ribonucleoprotein antibody; anti-SS-A, anti-Sjögren’s-syndrome-related antigen A autoantibody; anti-SS-B, anti-Sjögren’s-syndrome-related antigen B autoantibody; ANCA, antineutrophil cytoplasmic antibody.

**Table 7:** Comparison of renal pathological and clinical features, treatment, renal responses, and outcomes of ISN/RPS Class IV LN patients with other classes of LN patients.

	All Classes (*n* = 114) ^a^	Class I-II (*n* = 7)	Class III (*n* = 13)	Class V (*n* = 20)	Class IV (*n* = 74)	*p*-Value ^b^
**Renal pathological and clinical features**						
AI (*n* = 73), score ^c^	10 (6–12)	4 (2–4)	8 (6–8)	6 (4–10)	10 (8–14)	<0.001 *
CI (*n* = 73), score ^c^	3 (2–4)	0 (0–1.5)	3 (2–3)	2 (1–2)	3 (2–4)	<0.001 *
CKD stage 3–5	32 (28.1)	2 (28.6)	0 (0)	4 (20.0)	26 (35.1)	0.022 *
CKD stage 5 (ESRD)	11 (9.6)	0 (0)	0 (0)	2 (10.0)	9 (12.2)	0.324
**Treatment and renal responses**						
Steroid (currently)	73 (64.0)	4 (57.1)	8 (61.5)	13 (65.0)	48 (64.9)	0.802
Chloroquine	53 (46.5)	3 (42.9)	5 (38.5)	9 (45.0)	36 (48.6)	0.530
AZA	85 (74.6)	6 (85.7)	12 (92.3)	11 (55.0)	56 (75.7)	0.710
Maintenance Th	73 (64.0)	4 (57.1)	8 (61.5)	8 (40.0)	53 (71.6)	0.022 *
MMF						
Induction Th	48 (42.1)	3 (42.9)	4 (30.8)	10 (50.0)	31 (41.9)	0.950
Maintenance Th	33 (28.9)	1 (14.3)	2 (15.4)	8 (40.0)	22 (29.7)	0.802
CYC	94 (82.5)	6 (85.7)	11 (84.6)	15 (75.0)	62 (83.8)	0.612
MTX	12 (10.5)	1 (14.3)	3 (23.1)	1 (5.0)	7 (9.5)	0.751
CsA	12 (10.5)	0 (0)	1 (7.7)	7 (35.0)	4 (5.4)	**0.024m ^#^**
Tacrolimus	11 (9.6)	0 (0)	0 (0)	3 (15.0)	8 (10.8)	0.744
Plasmapheresis	24 (21.1)	2 (28.6)	4 (30.8)	3 (15.0)	15 (20.3)	0.780
Rituximab	16 (14)	2 (28.6)	2 (15.4)	1 (5.0)	11 (14.9)	0.729
Belimumab	8 (7.0)	0 (0)	1 (7.7)	2 (10.0)	5 (6.8)	1.000
CD, mg/kg	18,980 (8760–35,040)	18,980 (13,270–27,740)	29,200 (14,600–48,180)	21,170 (7545–29,930)	16,790 (8000–35,040)	0.587
CR	81 (71.1)	6 (85.7)	10 (76.9)	14 (70.0)	51 (68.9)	0.494
PR	17 (14.9)	1 (14.3)	2 (15.4)	4 (20.0)	10 (13.5)	0.568
NR	16 (14.0)	0 (0)	1 (7.7)	2 (10.0)	13 (17.6)	0.140
**Outcomes**						
SDI, score	1 (0–2)	1 (0–1.5)	1 (0–1)	1 (0–3)	1 (0–2)	0.333
LLDAS	23 (20.2)	2 (28.6)	1 (7.7)	5 (25.0)	15 (20.3)	0.973
Remission	18 (15.8)	2 (28.6)	4 (30.8)	2 (10.0)	10 (13.5)	0.365
Mortality	18 (15.8)	1 (14.3)	4 (30.8)	1 (5.0)	12 (16.2)	0.865
Cardiovascular	5 (27.8)	0 (0)	0 (0)	1 (100)	4 (33.3)	0.615
Infection	3 (16.7)	0 (0)	0 (0)	0 (0)	3 (25)	0.515
Sepsis	5 (27.8)	1 (100)	0 (0)	0 (0)	4 (33.3)	0.615
Tumour	3 (16.7)	0 (0)	3 (75.0)	0 (0)	0 (0)	**0.025 ^#^**
Underlying disease	2 (11.1)	0 (0)	1 (25.0)	0 (0)	1 (8.3)	1.000

Values are presented as frequency (%) or median (IQR), while *p*-values are calculated by Pearson’s chi-squared test or Fisher’s exact test and Mann–Whitney U test. Statistically significant variables (*p* < 0.05) are highlighted as bold text. ^a^ Total number of LN patients is 127, of which 11 patients had Mixed LN Class (Class I-II + III: 1 patient; Class I-II + IV: 1 patient; Class I-II + V: 1 patient; Class III + IV: 2 patients; Class III + V: 5 patients; Class IV + V: 1 patient), and 2 patients had Not Classified LN. These two groups were not analysed in this case. ^b^ Class IV vs. I-II + III + V Classes together. ^c^ Total number of LN patients is 114, of which 73 patients had data about LN Activity and Chronicity Index. The calculation of these indices was introduced for patients who were diagnosed after 2005. * Significantly higher in Class IV. **^#^** Significantly lower in Class IV. Abbreviations: LN, lupus nephritis; ISN/RPS, International Society of Pathology/Renal Pathology Society; *n*, number of patients; AI, activity index; CI, chronicity index; CKD, chronic kidney disease; ESRD, end-stage renal disease; AZA, azathioprine; MMF, mycophenolate mofetil; Th, therapy; CYC, cyclophosphamide; MTX, methotrexate; CsA, cyclosporine A; CD, cumulative dose; CR, complete remission; PR, partial remission; NR, no remission; SDI, SLICC/ACR Damage Index, LLDAS, lupus low-disease-activity state.

**Table 8:** Comparison of disease characteristics between renal responses in patients with LN (*n* = 127).

	CR (*n* = 91)	PR (*n* = 19)	*p*-Value CR vs. PR	NR (*n* = 17)	*p*-Value CR vs. NR
**Demographics**					
Sex (female)	82 (90.1)	19 (100)	0.354	13 (76.5)	0.122
Age, years	46 (38–54)	39 (29–45)	0.004 *	49 (44–52)	0.505
Age at onset of SLE, years	27 (21–35.5)	23 (19–30.5)	0.330	31 (22–35)	0.658
Duration of SLE, years	17 (12–24)	10 (5.5–16.5)	0.002 *	16 (12–24)	0.913
**Renal pathological findings and features**					
ISN/RPS LN Class					
I-II	6 (6.6)	1 (5.3)	1.000	0 (0)	0.587
III	10 (11)	2 (10.5)	1.000	1 (5.9)	1.000
IV	51 (56)	10 (52.6)	0.785	13 (76.5)	0.116
V	14 (15.4)	4 (21.1)	0.510	2 (11.8)	1.000
Mixed Class LN^a^	9 (9.9)	1 (5.3)	1.000	1 (5.9)	1.000
No Class LN ^a^	1 (1.1)	1 (5.3)	0.317	0 (0)	1.000
AI (*n* = 83), score ^b^	8.5 (6.5–11.5)	10 (3–15)	0.854	10 (6–12)	0.932
CI (*n* = 83), score ^b^	2 (2–3)	3 (2–3.5)	0.319	5 (4–6)	**<0.001 ^#^**
CKD stage 3–5	16 (17.6)	4 (21.1)	0.747	15 (88.2)	**<0.001 ^#^**
CKD stage 5 (ESRD)	0 (0)	1 (5.3)	0.173	11 (64.7)	**<0.001 ^#^**
**Serology**					
Anti-ß2-GP-1	46 (50.5)	10 (52.6)	0.869	11 (64.7)	0.283
Anti-CL	61 (67)	12 (63.2)	0.745	10 (58.8)	0.513
LA	16 (17.6)	2 (10.5)	0.734	5 (29.4)	0.316
Anti-dsDNA	87 (95.6)	18 (94.7)	1.000	15 (88.2)	0.239
Anti-Sm	35 (38.5)	10 (52.6)	0.253	7 (41.2)	0.833
Anti-ENA	52 (57.1)	10 (52.6)	0.718	10 (58.8)	0.898
Anti-RNP	28 (30.8)	9 (47.4)	0.164	9 (52.9)	0.077
Anti-SS-A (Ro)	55 (60.4)	11 (57.9)	0.837	11 (64.7)	0.740
Anti-SS-B (La)	35 (38.5)	6 (31.6)	0.573	10 (58.8)	0.118
ANCA	13 (14.3)	2 (10.5)	1.000	2 (11.8)	1.000
Cryoglobulin	5 (5.5)	0 (0)	0.585	2 (11.8)	0.303
Coombs test (+)	10 (11)	5 (26.3)	0.133	5 (29.4)	0.059
**Treatment**					
Steroid (currently)	54 (59.3)	18 (94.7)	**0.003 ^#^**	9 (52.9)	0.623
Chloroquine	46 (50.5)	7 (36.8)	0.277	7 (41.2)	0.478
AZA	72 (79.1)	13 (68.4)	0.368	11 (64.7)	0.217
Maintenance Th	62 (68.1)	11 (57.9)	0.390	8 (47.1)	0.095
MMF					
Induction Th	29 (31.9)	12 (63.2)	**0.010 ^#^**	11 (64.7)	**0.010 ^#^**
Maintenance Th	22 (24.2)	9 (47.4)	**0.041 ^#^**	4 (23.5)	1.000
CYC	71 (78)	16 (84.2)	0.759	16 (94.1)	0.185
MTX	9 (9.9)	1 (5.3)	1.000	2 (11.8)	0.683
CsA	10 (11)	2 (10.5)	1.000	1 (5.9)	1.000
Tacrolimus	5 (5.5)	4 (21.1)	**0.046 ^#^**	3 (17.6)	0.110
Plasmapheresis	17 (18.7)	4 (21.1)	0.757	5 (29.4)	0.332
Rituximab	11 (12.1)	5 (26.3)	0.148	1 (5.9)	1.000
Belimumab	3 (3.3)	5 (26.3)	**0.004 ^#^**	0 (0)	1.000
CD, mg/kg	14,600 (7300–31,390)	11,680 (6085–30,295)	0.618	21,900 (11,680–37,960)	0.344
**Outcomes**					
SDI, score	0 (0–1)	0 (0–1)	0.346	3 (1–3)	**<0.001 ^#^**
LLDAS	17 (18.7)	0 (0)	0.040 *	6 (35.3)	0.192
Remission	19 (20.9)	1 (5.3)	0.188	2 (11.8)	0.517
Mortality	13 (14.3)	2 (10.5)	1.000	3 (17.6)	0.720
Cardiovascular	5 (38.5)	0 (0)	0.524	0 (0)	0.509
Infection	2 (15.4)	0 (0)	1.000	1 (33.3)	0.489
Sepsis	4 (30.8)	0 (0)	1.000	1 (33.3)	1.000
Tumour	1 (7.7)	1 (50)	0.257	1 (33.3)	0.350
Underlying disease	1 (7.7)	1 (50)	0.257	0 (0)	1.000

Values are presented as frequency (%) or median (IQR), while *p*-values are calculated by Pearson’s chi-squared test or Fisher’s exact test and Mann–Whitney U test. Statistically significant variables (*p* < 0.05) are highlighted as bold text. ^a^ Total number of LN patients is 127, of which 11 patients had Mixed LN Class (Class I-II + III: 1 patient; Class I-II + IV: 1 patient; Class I-II + V: 1 patient; Class III+IV: 2 patients; Class III + V: 5 patients; Class IV + V: 1 patient), and 2 patients had Not Classified LN. ^b^ Total number of LN patients is 127, of which 83 patients had data about LN Activity and Chronicity Index. The calculation of these indices was introduced for patients who were diagnosed after 2005. * Significantly more common in the CR group (CR vs. PR; CR vs. NR). **^#^** Significantly less common in the CR group (CR vs. PR; CR vs. NR). Abbreviations: LN, lupus nephritis; n, number of patients; CR, complete remission; PR, partial remission; NR, no remission; ISN/RPS, International Society of Pathology/Renal Pathology Society; AI, activity index; CI, chronicity index; CKD, chronic kidney disease; ESRD, end-stage renal disease; anti-ß2GPI, anti-β2-glycoprotein-1 antibody; anti-CL, anticardiolipin antibody; LA, lupus anticoagulant; anti-dsDNA, anti-double-stranded DNA; anti-Sm, anti-Smith antibody; anti-RNP, anti-ribonucleoprotein antibody; anti-SS-A, anti-Sjögren’s-syndrome-related antigen A autoantibody; anti-SS-B, anti-Sjögren’s-syndrome-related antigen B autoantibody; ANCA, antineutrophil cytoplasmic antibody; AZA, azathioprine; MMF, mycophenolate mofetil; Th, therapy; CYC, cyclophosphamide; MTX, methotrexate; CsA, cyclosporine A; CD, cumulative dose; SDI, SLICC/ACR Damage Index, LLDAS, lupus low-disease-activity state.

## Data Availability

The original contributions presented in the study are included in the article. Further inquiries can be directed to the corresponding author.
